# Decrease in Serum Vitamin D Level of Older Patients with Fatigue

**DOI:** 10.3390/nu11102531

**Published:** 2019-10-20

**Authors:** Manuela Pennisi, Giulia Malaguarnera, Giuseppe Di Bartolo, Giuseppe Lanza, Rita Bella, Eleonora Margherita Chisari, Omar Cauli, Enzo Vicari, Michele Malaguarnera

**Affiliations:** 1 Department of Biomedical and Biotechnological Science, University of Catania, 95125 Catania, Italy; manuela.pennisi@unict.it (M.P.); giulia.malaguarnera@live.it (G.M.); 2 Research Center “The Great Senescence”, University of Catania, 95100 Catania, Italy; Gdiba9@gmail.com; 3 Department of Surgery and Medical-Surgical Specialties and Advanced Technology, Section of Neurosciences, University of Catania, 95125 Catania, Italy; glanza@oasi.en.it; 4 Department of Neurology IC, Oasi Research Institute—IRCCS, 94018 Troina, Italy; 5 Department of Medical and Surgical Sciences and Advanced Technology, Section of Neurosciences, University of Catania, 95125 Catania, Italy; rbella@unict.it; 6 Department of Education, University of Catania, I-95124 Catania, Italy; g.chisari@tin.it; 7 Department of Nursing, University of Valencia, 46010 Valencia, Spain; omar.cauli@uv.es; 8 Department of Clinical and Experimental Medicine, University of Catania, 95123 Catania, Italy; enzodante@email.it

**Keywords:** vitamin D, older, aging, physical fatigue, mental fatigue, sex differences

## Abstract

Fatigue is characterized by reduced energy level, decreased muscle strength, and a variable degree of cognitive impairment. Recent evidences seem to link vitamin D deficiency to fatigue. The aim of this study was to assess and compare vitamin D status in a cohort of older subjects with and without fatigue. We recruited a total of 480 subjects, 240 patients with fatigue and 240 controls without fatigue, from the Cannizzaro Hospital of Catania (Italy). Fatigue severity was measured by the fatigue severity scale, whereas mental and physical fatigue were measured through the Wessely and Powell fatigue scale, respectively. We also measured several blood parameters and 25-OH vitamin D. Subjects with fatigue showed lower levels of vitamin D as compared with those without fatigue. Blood levels of parameters related to fatigue were normal in both groups of subjects, however, platelet, hemoglobin, hematocrit (*p* < 0.05), mean corpuscular volume, C-reactive protein (CRP), iron, vitamin B12, and folic acid (*p* < 0.001) were significantly higher in the fatigue group with respect to the control group. Moreover, compared to controls, patients showed higher scores in the physical (*p* < 0.001), mental (*p* < 0.001), and severity (*p* < 0.001) fatigue scales. Finally, vitamin D inversely correlated with fatigue severity (*r* = −0.428, *p* < 0.01), whereas creatine kinase and CRP levels did not correlate with vitamin D. In conclusion, our data showed a direct link between vitamin D and fatigue in older subjects, suggesting translational implications in the diagnosis and management of these patients.

## 1. Introduction

Fatigue is a complex nonspecific clinical condition characterized by a difficulty in initiating or sustaining voluntary activities, a mismatch between the effort and the actual performance, an overwhelming feeling of tiredness at rest, an exhaustion with activity, a lack of energy that impairs daily tasks, and an inertia, with loss of endurance and vigor [1]. “The Diagnostic and Statistical Manual of Mental Disorders”, fifth edition defines fatigue as a “state usually associated with a weakening or depletion of one’s physical and or mental resources ranging from a general state of lethargy to specific work-induced burning sensation within one’s muscle.”

Most frequently, although it represents a response to both physical and mental exercise or stress, or a sign of some illnesses such as infection, inflammation, cancer, and autoimmune disease, fatigue is commonly reported in older subjects often without a clear underlying cause [2]. Physical fatigue leads to the inability to continue the individual functioning at a normal level of activity. Conversely, although it is widespread during heavy exercise, mental fatigue often manifests as somnolence [3]. Overall, fatigue can cause significant impairment of quality of life (QoL), with an estimated annual cost of 126 billion of US Dollars among USA employers [4]. Fatigue is also a common complaint in primary health care departments of both developed and developing countries, with a relevant impact on social life, family activities, and work performance [5,6,7].

One of the biological factors that has been linked to fatigue is vitamin D deficiency [8,9]. Vitamin D refers to a group of fat-soluble secosteroid hormones ingested through various dietary sources [10]. It is known that sun exposure activates vitamin D constituents, which improves health status and biological immune responses. Vitamin D deficiency is considered to be a common condition, especially among the elders suffering from muscular pain, headache, weakness, osteomalacia, sarcopenia, and other age-related problems [11]. Indeed, the Second National Report on the Biochemical Indicators of Diet and Nutrition in the USA stated that 25.5% of the older population, studied from 2003 to 2006, showed vitamin D deficiency (52.5–72.5 nmol/L) and that 8.8% had low serum 25-hydroxyvitamin D concentration (<50 nmol/L) [12,13].

Vitamin D is also involved in several biological processes through its specific receptor (Vitamin D Receptor - VDR) [14] and is also necessary for bone and skeletal muscle metabolism. Therefore, its deficiency has been associated with musculoskeletal pain [15], chronic pain [16], low back pain [17], bone disorders, myopathy [18,19], immune dysregulation, and daytime sleepiness [20]. Additionally, studies on rats have found that serum vitamin D deficiency plays a role in the etiology of deep muscle pain and that its supplementation can decrease the levels of plasma creatine kinase (CK) and some proinflammatory cytokines by reducing the gene expression of interleukin-6 (IL-6) and tumor necrosis factor-alpha (TNF-α) [21,22]. In this framework, recent studies have demonstrated that obesity might be strongly associated with low-grade chronic inflammation, which is accompanied by raised concentrations of inflammatory cytokines, including IL-6 and TNF-α, and acute phase proteins, such as C-reactive protein (CRP). Similarly, higher plasma concentrations of proinflammatory cytokines have been found in subjects with low serum vitamin D. The mechanism by which vitamin D influences inflammatory biomarkers is not well known, although some authors propose the role of adipokines as a possible link between vitamin D status and obesity [23]. In vitro studies showed that vitamin D might inhibit the production of IL-6, and some investigators support a model in which calcium and vitamin D treatment directly and indirectly blocks the TNF-α pathway [22,23]. Finally, an inverse relationship between TNF-α levels and serum 25-hydroxyvitamin D has been demonstrated [24].

Nevertheless, although the relationship between vitamin D and fatigue seems to be established in clinical practice, uncertainty still remains whether this association is causal or not. The aim of the present study is to evaluate and compare vitamin D status between subjects with and without fatigue in a cohort of older subjects.

## 2. Materials and Methods

### 2.1. Subjects

Participants were enrolled from the “Osteoporosis Prevention Center” of the Cannizzaro Hospital of Catania (Italy), from January 2014 to December 2017. In total, we screened 510 older outpatients, 249 with fatigue and 251 without fatigue. Among them, we eventually included in the study 240 subjects with fatigue (patients, mean age 69.1 ± 5.8 years) and 240 without fatigue (controls, mean age 69.2 ± 5.1 years). Twenty subjects were excluded due to the following reasons: 2 were affected by type 2 diabetes, with a glycosylated hemoglobin level of 9%; 1 was on antihistamine drug; 4 did not completed the questionnaires; and 13 did not provide the written consent to participate in the study. All subjects were submitted to the fatigue test scores and subsequently to blood analysis. They were instructed to not change their normal eating habits during the entire period of data collection.

The inclusion criteria were as follows: (1) older than 65 years, (2) fatigue for more than 4 weeks and stable chronic medical condition, (3) absence of severe psychiatric or cognitive disorder, and (4) ability to understand and complete the questionnaires. Subjects were excluded in case of the following: (1) chronic infectious diseases, pulmonary disorders, and depression; (2) anemia and others hematological diseases; (3) dehydration; (4) advanced neurological diseases associated with fatigue, such as multiple sclerosis; (5) advanced cardiac diseases with poor performance status; (6) uncontrolled endocrinological disorders (e.g., hypothyroidism, hyperthyroidism, diabetes with glycosilated hemoglobin >9%); (7) advanced kidney or liver disease (e.g., cirrhosis); (8) active or advanced cancer or other neoplasms; (9) autoimmune diseases; (10) current intake benzodiazepines, neuroleptic, antiepileptic drugs, antihistamines, narcotics, corticosteroids, diuretics, and HMG-CoA reductase inhibitors (i.e., statins); (11) previous treatment with vitamin D; and (12) a vegan or vegetarian diet.

All the recruited participants gave a written consent after being fully informed on the study and completed a prepared self-administrated questionnaire. The study was approved by the Institutional Review Board of the Research Center “The Great Senescence” based at the Cannizzaro Hospital of Catania (Italy).

### 2.2. Questionnaire Evaluating Fatigue

All demographic and clinical data were collected and recorded by trained operators. Weight and height were measured without shoes. Body mass index (BMI) was calculated by the height squared in meters (Kg/m^2^). The participants also completed a self-reported questionnaire.

Fatigue severity was measured by the fatigue severity scale (FSS). The FSS is a self-assessed nine-question scale, each item ranging from 1 (strongly disagree with the statement) to 7 (maximum agreement). Here, the total score ranged from 9 to 63, and it was directly related to the severity observed on the nine-item fatigue questionnaire. [25]. The Wessely’s test and Powell’s test were used to examine mental and physical fatigue, respectively. Their scores consisted of a total list of 14 questions, divided in two scales measuring physical fatigue (8 items scored from 0 (no fatigue) to 2 (highest possible fatigue), total score range 0–16); and mental fatigue (5 items, total score range 0–10) [26,27]. These scales have been previously used and validated in older populations [28,29].

### 2.3. Laboratory Measurements

Biological samples were collected during the administration of the fatigue questionnaires. Venous blood samples were collected after overnight fasting.

We used an automatic biochemical analyzer to measure albumin, gamma-glutamyl-transferase (γGT), creatinine, calcium, phosphorus, white blood cells (WBC), platelets (PLT), red blood cells (RBC), hemoglobin (HGB), hematocrit (HCT), mean corpuscular volume (MCV), mean corpuscular hemoglobin (MCH), red blood cells distribution width (RDW) iron, vitamin B12, folic acid, and total bilirubin. Commercially available kits were used to estimate serum calcium level by calorimetric assay. We determined serum alanine aminotransferase (ALT) and aspartate aminotransferase (AST) using an enzymatic calorimetric test. CRP was measured using the high sensitivity nephelometric method. Serum CK was assessed by a standardized commercially available enzymatic assay. Regarding the measurement of serum 25-OH vitamin D, blood samples were obtained from study subjects from June 2014 to December 2017, and serum vitamin D levels by immunoenzymatic assay (Beckmann Coulter).

Participants were instructed to fast for at least 12 hours before analysis. Fasting blood samples were collected in the morning, immediately centrifugated, and the supernatant transferred to new tube. The intra-assay coefficient of variation (CV) was 29% and the inter-assay CV was 3.2%.

### 2.4. Statistics Analysis

All data management and statistical calculations were performed using SPSS 15.0 statistical package (Chicago, IL, USA). The categorical variables were expressed as proportions and the numerical variables as means ± standard deviations or as medians with interquartile ranges. The comparison between the study groups was performed by using the t-test, Mann–Whitney U test, or the Chi-square, as appropriate. Correlations were determined according to the Pearson’s method. *p* values < 0.05 were considered as statistically significant.

## 3. Results

In total, we examined 480 older subjects using the abovementioned fatigue assessment scales, those complaining of fatigue (240) and those who did not complain of fatigue (240). Participants’ baseline characteristics are summarized in Table 1.

Serum analyses (Table 2) showed normal values for the following parameters: WBC, RBC, and RDW. The other measurements, namely, PLT, HGB, HCT (*p* < 0.05), MCV, CRP, iron, vitamin B12, and folic acid (*p* < 0.01), although still within normal ranges, were significantly higher in the fatigue group than in the control group. The MCH and phosphorus levels were significantly lower for the fatigue group (*p* < 0.001) (Table 2). Finally, compared with controls, subjects with fatigue showed a significant decrease in vitamin D level (*p* < 0.001).

At the fatigue rating scales, we found significantly higher scores in the physical (*p* < 0.0001), mental (*p* < 0.0001), and severity (*p* < 0.0001) fatigue scales in patients than controls (Table 3).

Additionally, in patients with fatigue, we observed a significant intra-group gender difference in terms of worse scores in males than females for the physical fatigue (1.1, *p* < 0.01), mental fatigue (0.7, *p* < 0.01), and fatigue severity scores (4.0, *p* < 0.01). Conversely, in controls, we did not find a difference in physical fatigue score, although males performed worse in mental fatigue (0.5, *p* < 0.05) and fatigue severity scores (3.6, *p* < 0.01) (Table 4). Compared to female controls, those with fatigue showed a significant difference in physical fatigue (11.0 vs. 6.7, *p* < 0.01), mental fatigue (6.4 vs. 5.1, *p* < 0.01), and fatigue severity scores (44.2 vs. 28.2, *p* < 0.01). Vitamin D level was significant lower in females with fatigue than controls (39.2 vs. 46.9 nmol/L, *p* < 0.01). Similar results were observed for the male participants with fatigue in terms of worse scores in physical fatigue (12.1 vs. 7.1, *p* < 0.01), mental fatigue (7.1 vs. 5.6, *p* < 0.01), and fatigue severity scores (48.2 vs. 31.8, *p* < 0.01) as compared with those without. Moreover, males with fatigue exhibited lower vitamin D serum level as compared with control males (40.2 vs. 48.2 nmol/L, *p* < 0.01).

Finally, vitamin D serum levels were higher in patients than controls (*p* < 0.001). Vitamin D level also inversely correlated with fatigue severity score (*r* = −0.428, *p* < 0,01), whereas CK and CRP did not correlate.

## 4. Discussion

### 4.1. Main Findings

Fatigue is a common clinical condition characterized by decreased energy, concentration, and motivation that interferes with the activities of daily living. As such, fatigue is also associated with several physical and psychological symptoms, including pain, depression, distress, dyspnea, anxiety, and poor sleep quality [30]. Patients with an induced fatigue state show a reduced exercise tolerance and a post-exercise fatigue even after minimal physical activity, thus, suggesting that an impaired muscle function may occur behind this condition.

**Figure 1:**
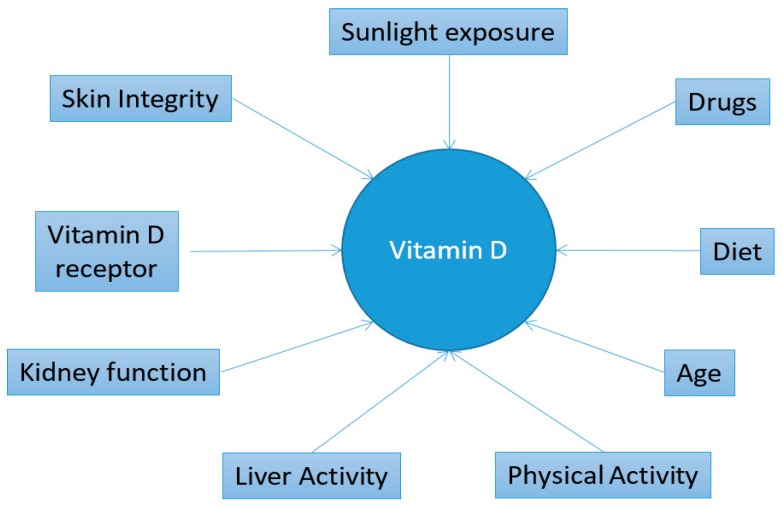
Factors influencing vitamin D levels.

In our study, we observed a significant reduction of vitamin D serum level in older subjects with fatigue. This result is in agreement with a prospective nonrandomized therapeutic study in USA general population. Moreover, the authors showed that normalization of vitamin D level was able to improve the severity of fatigue [8]. In a preliminary report, it has also been demonstrated that vitamin D supplementation improved symptoms of fibromyalgia, including fatigue [31]. By contrast, another study on chronic fatigue syndrome/myalgic encephalomyelitis revealed no association between perceived fatigue and vitamin D levels [32]. It has also been reported that vitamin supplementation may not affect fatigue on frailty older patients [33]. Indeed, supplementation is not always able to reduce fatigue. As illustrated in Figure 1, this effect might be explained by several factors associated with vitamin D presence, such as reduced vitamin dietary intake, limited sun exposure, and renal dysfunction. Moreover, multiple factors linked to the fatigue status may also contribute. For instance, some RBC indices (i.e., RDW, MCV, and MCH) have been related to fatigue. RBC are of pivotal importance in the human energy dynamic, and, it is known that they need to deform themselves to travel through capillaries for effective oxygen transportation. RBC deformability has been shown to be affected by ALT [34], AST [35], CRP [36], and 25-OH vitamin D levels [37]. Interestingly, recently, RBC deformability has been found to be impaired also in patients with chronic fatigue syndrome [38].

In older subjects, the ability of the skin to synthesize the vitamin D decreases substantially. Accordingly, compared to 20-year-old individuals, subjects at 70 years of age reduce their vitamin D levels by more than 50% due to decreased cutaneous synthesis [39]. In Europe, in older people, vitamin D deficiency occurs more frequently in women, and more commonly in the south than in the north countries [40]. Although many studies found low vitamin D level in large proportions of older people (but most of them used a limit range of 20–25 nmol/L [41,42,43,44,45]), the exact limit of vitamin D3 delineating vitamin D deficiency is still debated. In this scenario, in agreement with previous findings, we found that vitamin D was significantly lower in the fatigue group as compared with the control group. Additionally, although values still were within the normal limits, patients with fatigue showed significantly different levels of CRP, phosphorus, PLT, HCT, MCV, MCH, RBC, iron, vitamin B12, and folic acid as compared with controls. Thus, vitamin D might not be the only factor affecting the feeling of fatigue of these patients, however, a low level of vitamin D may contribute to the wide spectrum of fatigue-related clinical manifestations. Indeed, low levels of vitamin D can significantly impact both physical and mental well-being [10,46,47]. Risk factors contributing to vitamin D deficiency in older adults include reduced nutritional intake, increasing adiposity, decreased cutaneous synthesis, and less time spent outdoor [48].

Vitamin D deficiency has been associated with reduced muscle strength, physical performance, postural stability, and QoL. In particular, the growing awareness that fatigue is a major contributor affecting QoL has led to a significant effort to obtain further data on mental and physical tiredness, also with therapeutic implications. In some observational studies, the reverse causality is relevant, as low vitamin D status may be considered a marker or a consequence of fatigue, however, the association between vitamin D and fatigue might also reflect the presence of confounding factors associated with vitamin deficiency and fatigue status [49]. In fact, both are associated with a range of lifestyle-related features, including low educational attainment, smoking, and physical inactivity [50,51,52]. In this context, it is worth mentioning that exercise-induced tissue damage and lipid peroxidation have been significantly lowered by vitamin D treatment in severely deficient human subjects [53]. Finally, the low serum levels of vitamin D have been associated with high fatigue severity in older subjects [54]. Therefore, based on our findings and considerations from results in the literature, the threshold level of vitamin D deficiency should be reconsidered in older patients.

Finally, we found some gender-related differences in fatigue scales and vitamin D, both in patients and controls. The explanation for these findings is rather complex and probably is associated with the distinctive biochemical and hormonal aspects related to the vitamin D molecular structure and metabolism. In particular, the molecular structure of vitamin D is closely allied to that of typical steroid hormones (e.g., estradiol, cortisol, testosterone, and aldosterone). Moreover, impaired 25-hydroxylation and 1α-hydroxylation by liver or renal disease, as well as genetic causes or mutated nonfunctioning VDR, might explain these gender differences. It is worth highlighting that although vitamin D deficiency or insufficiency is highly prevalent its magnitude may largely vary depending on the population studied, geographical areas, and seasonal considerations [55,56].

### 4.2. Limitations

One limitation, in this study, is that fatigue prevalence, severity, and related socioeconomic factors were not considered. Another limitation is based on the single center nature of the results, thus, precluding any further generalization of our findings. Indeed, data were limited to an initial single serum 25-hydroxyvitamin D measurement at the time of enrolment. Therefore, we acknowledge that this measurement could not reflect long-lasting periods of vitamin D level. As mentioned, geographical locations determine marked differences in dietary vitamin D intake and, more importantly, in sun exposure, which can greatly influence serum vitamin concentration in the general population. Finally, the cross-sectional nature of the present study does not allow any causality interpretation. Overall, given these limitations and critical aspects, further research is needed to better understand the biochemical mechanisms underlying the results provided in this study.

## 5. Conclusions

Vitamin D deficiency was associated with mental and physical fatigue, suggesting that supplementation may be helpful in reducing the risk of fatigue in older people. Further larger studies and multidimensional follow-up are needed to confirm these findings. 

## Figures and Tables

**Table 1:** Patients’ baseline characteristics.

Variable	Patients	Controls	*p*
Age range, years (mean ± SD)	69.10 ± 5.80	69.20 ± 5.10	/
Heart rate, bpm (mean ± SD)	82.80 ± 8.20	81.80 ± 8.60	NS
Systolic blood pressure, mmHg (mean ± SD)	140.00 ± 9.10	138.20 ± 9.60	<0.001
Diastolic blood pressure, mmHg (mean ± SD)	79.00 ± 7.50	79.20 ± 7.20	NS
Body Mass Index, Kg/m^2^ (mean ± SD)	24.80 ± 3.40	24.40 ± 3.20	NS
Current/Former smoker (%)	38.70	37.91	/
Diabetes Mellitus (%)	9.16	10.00	/
Hypertension (%)	17.08	17.50	/
Heart insufficiency (%)	4.58	4.16	/
Education, no Diploma (%)	48.33	42.91	/
Education. High School Diploma (%)	30.41	30.41	/
Education, University Degree (%)	20.83	14.16	/

bpm: beats per minute; NS: not significant; SD: standard deviation.

**Table 2:** Laboratory measurement on patients’ sera.

Variable	Normal Range	Patients	Controls	*p*
CK, IU/L	10.00–171.00	36.70 ± 11.80	37.00 ± 11.40	NS
Bilirubin, mg/dl	0.30–1.20	0.80 ± 0.10	0.81 ± 0.10	NS
Albumin, g/dl	3.50–4.80	4.20 ± 0.35	4.20 ± 0.31	NS
AST, IU/L	5.00–45.00	36.00 ± 7.20	35.00 ± 6.80	NS
ALT, IU/L	5.00–45.00	37.10 ± 9.00	36.50 ± 9.20	NS
γGT, IU/L	5.00–55.00	33.20 ± 8.60	32.10 ± 9.70	NS
Creatinine, mg/dL	0.50–1.10	0.93 ± 0.20	0.91 ± 0.23	NS
Vitamin D, nmol/L (median; range)	75.00–200.00	39.50 ± 11.80 (41.60; 25.00–51.00)	48.10 ± 13.80 (60.20; 36.00–68.00)	<0.001
CRP, mg/L (median; range)	0.00–4.80	4.31 ± 1.70 (3.96; 1.60–5.20)	1.44 ± 1.10 (1.30; 0.50–2.00)	<0.001
Calcium, mg/dl	8.10–10.10	8.50 ± 1.90	8.40 ± 2.00	NS
Phosphorus, mg/dl	2.50–4.50	2.40 ± 0.70	2.90 ± 0.70	<0.001
WBC, 10^3^ U/L	4.00–9.50	6.70 ± 1.20	6.50 ± 1.30	NS
PLT, 10^3^ U/L	150.00–410.00	304.00 ± 26.00	298.00 ± 32.00	<0.05
RBC, 10^6^ U/L	4.50–6.10	4.64 ± 0.82	4.52 ± 0.91	NS
HGB, g/dl	13.00–18.00	14.20 ± 0.70	14.00 ± 0.87	<0.05
HCT, %	38.00–50.00	44.10 ± 0.60	43.90 ± 0.80	<0.05
MCV, fL	80.00–102.00	94.10 ± 0.80	93.80 ± 0.90	<0.001
MCH, pg	26.00–33.00	30.20 ± 0.80	30.60 ± 0.70	<0.001
RDW, %	9.80–16.00	14.10 ± 1.80	13.80 ± 2.00	NS
Iron, µg/dL	65.00–170.00	87.40 ± 11.80	82.60 ± 13.60	<0.001
Vitamin B12, pg/dL	180.00–914.00	424.20 ± 26.80	412.80 ± 31.90	<0.001
Folic acid, ng/dL	3.10–19.90	8.78 ± 0.34	8.61 ± 0.42	<0.001

CK: creatine kinase; AST: aspartate aminotransferase; ALT: alanine aminotransferase; γGT: gamma-glutamyl-transferase; CRP: C-reactive protein; WBC: white blood cells; PLT: platelets; RBC: red blood cells; HGB: hemoglobin; HCT: hematocrit; MCV: mean corpuscular volume; MCH: mean corpuscular hemoglobin; NS: not significant; RDW: red blood cells distribution width.

**Table 3:** Fatigue questionnaires data from subjects with and without fatigue.

Scale (mean ± SD)	Patients	Controls	*p*
Physical fatigue scale score	11.5 ± 1.2	6.9 ± 1.3	<0.0001
Mental fatigue scale score	6.7 ± 1.8	5.3 ± 1.2	<0.0001
Fatigue severity score	46.1 ± 3.2	29.5 ± 3.2	<0.0001

**Table 4:** Gender differences of fatigue scale scores and vitamin D levels between patients and controls.

Variable	Patients			Controls			Intergroup Comparison	
	F	M	*P* (M vs. F)	F	M	*P* (M vs. F)	*p* (Control F vs. Patient F)	*P* (Control M vs. Patient M)
Physical fatigue scale	11.0 ± 1.2	12.1 ± 1.3	<0.0001	6.7 ± 1.2	7.1 ± 1.4	0.0731	<0.0001	<0.0001
Mental fatigue scale	6.4 ± 1.8	7.1 ± 1.7	0.0017	5.1 ± 1.1	5.6 ± 1.2	0.0468	<0.0001	<0.0001
Fatigue severity scale	44.2 ± 3.2	48.2 ± 3.2	<0.0001	28.2 ± 3.1	31.8 ± 3.6	<0.0001	<0.0001	<0.0001
Vitamin D, nmol/L	39.2 ± 14.9	40.2 ± 12.8	NS	46.9 ± 21.4	48.2 ± 22.7	NS	0.0045	0.0075

F: female; M: male; NS: not significant; numbers in bold: statistically significant *p* value.

## References

[1] Chaudhuri A., Behan P.O. (2004). Fatigue in neurological disorders. Lancet.

[2] Meng H., Hale L., Friedberg F. (2010). Prevalence and Predictors of Fatigue among Middle-Aged and Older Adults: Evidence from the Health and Retirement Study. J. Am. Geriatr. Soc..

[3] Arlington V.A. (2013). Diagnostic and Statistical Manual of Mental Disorders: DSM-5TM.

[4] Bower J.E. (2012). Fatigue, brain, behavior, and immunity: Summary of the 2012 Named Series on fatigue. Brain Behav. Immun..

[5] Kroenke K., Arrington M.E., Mangelsdorff A.D. (1990). The prevalence of symptoms in medical outpatients and the adequacy of therapy. Arch. Intern. Med..

[6] Swain M.G. (2000). Fatigue in chronic disease. Clin. Sci..

[7] Huppertz-Hauss G., Høivik M.L., Jelsness-Jørgensen L.-P., Opheim R., Henriksen M., Høie O. (2017). Fatigue in a population-based cohort of patients with inflammatory bowel disease 20 years after diagnosis: The IBSEN study. Scand. J. Gastroenterol..

[8] Roy S., Sherman A., Monari-Sparks M.J., Schweiker O., Hunter K. (2014). Correction of Low Vitamin D Improves Fatigue: Effect of Correction of Low Vitamin D in Fatigue Study (EViDiF Study). N. Am. J. Med. Sci..

[9] Nowak A., Boesch L., Andres E., Battegay E., Hornemann T., Schmid C., Bischoff-Ferrari H.A., Suter P.M., Krayenbuehl P.A. (2016). Effect of vitamin D3 on self-perceived fatigue: A double-blind randomized placebo-controlled trial. Medicine.

[10] Holick M.F. (2007). Vitamin D deficiency. N. Engl. J. Med..

[11] Boccardi V., Lapenna M., Gaggi L., Garaffa F.M., Croce M.F., Baroni M., Ercolani S., Mecocci P., Ruggiero C. (2019). Hypovitaminosis D: A Disease Marker in Hospitalized Very Old Persons at Risk of Malnutrition. Nutrients.

[12] US Centers for Disease Control and Prevention (2012). Second National Report on Biochemical Indicators of Diet and Nutrition in the U.S. Population 2012.

[13] Holick M.F., Binkley N.C., Bischoff-Ferrari H.A., Gordon C.M., Hanley D.A., Heaney R.P., Murad M.H., Weaver C.M. (2011). Evaluation, treatment, and prevention of vitamin D deficiency: An Endocrine Society clinical practice guideline. J. Clin. Endocrinol. Metab..

[14] Stewart J.W., Alekel D.L., Ritland L.M., Van Loan M., Gertz E., Genschel U. (2009). Serum 25-hydroxyvitamin D is related to indicators of overall physical fitness in healthy postmenopausal women. Menopause.

[15] Plotnikoff G.A., Quigley J.M. (2003). Prevalence of Severe Hypovitaminosis D in Patients with Persistent, Nonspecific Musculoskeletal Pain. Mayo Clin. Proc..

[16] Turner M.K., Hooten W.M., Schmidt J.E., Kerkvliet J.L., Townsend C.O., Bruce B.K. (2008). Prevalence and clinical correlates of vitamin D inadequacy among patients with chronic pain. Pain Med..

[17] Lotfi A., Abdel-Nasser A.M., Hamdy A., Omran A.A., El-Rehany M.A. (2007). Hypovitaminosis D in female patients with chronic low back pain. Clin. Rheumatol..

[18] Goldstein M.R. (2007). Myopathy, statins, and vitamin D deficiency. Am. J. Cardiol..

[19] Bolton C.F. (2005). Neuromuscular manifestations of critical illness. Muscle Nerv..

[20] Hoeck A.D., Pall M.L. (2011). Will vitamin D supplementation ameliorate diseases characterized by chronic inflammation and fatigue?. Med. Hypotheses.

[21] Tague S.E., Clarke G.L., Winter M.K., McCarson K.E., Wright D.E., Smith P.G. (2011). Vitamin D Deficiency Promotes Skeletal Muscle Hypersensitivity and Sensory Hyperinnervation. J. Neurosci..

[22] Choi M., Park H., Cho S., Lee M. (2013). Vitamin D3 supplementation modulates inflammatory responses from the muscle damage induced by high-intensity exercise in SD rats. Cytokine.

[23] Jamka M., Woźniewicz M., Jeszka J., Mardas M., Bogdański P., Stelmach-Mardas M. (2015). The effect of vitamin D supplementation on insulin and glucose metabolism in overweight and obese individuals: Systematic review with meta-analysis. Sci. Rep..

[24] Peterson C.A., Heffernan M.E. (2008). Serum tumor necrosis factor-alpha concentrations are negatively correlated with serum 25(OH)D concentrations in healthy women. J. Inflamm..

[25] Krupp L.B., LaRocca N.G., Muir-Nash J., Steinberg A.D. (1989). The fatigue severity scale. Application to patients with multiple sclerosis and systemic lupus erythematosus. Arch. Neurol..

[26] Wessely S., Powell R. (1989). Fatigue syndromes: Acomparison of chronic postviral fatigue with neuromuscular affective disorders. J. Neurol. Neurosurg. Psychiatry.

[27] Chalder T., Berelowitz G., Pawlikowska T., Watts L., Wessely S., Wright D., Wallace E.P. (1993). Development of a fatigue scale. J. Psychosom. Med..

[28] Pistone G., Marino A., Leotta C., Dell’Arte S., Finocchiaro G., Malaguarnera M. (2003). Levocarnitine administration in elderly subjects with rapid muscle fatigue: Effect on body composition, lipid profile and fatigue. Drugs Aging.

[29] Malaguarnera M., Gargante M.P., Cristaldi E., Colonna V., Messano M., Koverech A., Neri S., Vacante M., Cammalleri L., Motta M. (2008). Acetyl L-carnitine (ALC) treatment in elderly patients with fatigue. Arch. Gerontol. Geriatr..

[30] Pennisi M., Di Bartolo G., Malaguarnera G., Bella R., Lanza G., Malaguarnera M. (2019). Vitamin D Serum Levels in Patients with Statin-Induced Musculoskeletal Pain. Dis. Markers.

[31] de Carvalho J.F., da Rocha Araújo F.A.G., da Mota L.M.A., Aires R.B., de Araujo R.P. (2018). Vitamin D Supplementation Seems to Improve Fibromyalgia Symptoms: Preliminary Results. Isr. Med. Assoc. J..

[32] Earl K.E., Sakellariou G.K., Sinclair M., Fenech M., Croden F., Owens D.J., Tang J., Miller A., Lawton C., Dye L. (2017). Vitamin D status in chronic fatigue syndrome/myalgic encephalomyelitis: A cohort study from the North-West of England. BMJ Open.

[33] Latham N.K., Anderson C.S., Lee A., Bennett D.A., Moseley A., Cameron I.D., Fitness Collaborative Group (2003). A randomized, controlled trial of quadriceps resistance exercise and vitamin D in frail older people: The Frailty Interventions Trial in Elderly Subjects (FITNESS). J. Am. Geriatr. Soc..

[34] Vayá A., Bonet E., Romagnoli M., Nuñez C., Todoli J. (2010). Erythrocyte deformability in macrocytosis determined by means of ektacytometry techniques. Clin. Hemorheol. Microcirc..

[35] Cita K.C., Brureau L., Lemonne N., Billaud M., Connes P., Ferdinand S., Tressières B., Tarer V., Etienne-Julan M., Blanchet P. (2016). Men with Sickle Cell Anemia and Priapism Exhibit Increased Hemolytic Rate, Decreased Red Blood Cell Deformability and Increased Red Blood Cell Aggregate Strength. PLoS ONE.

[36] Tsuda K. (2012). Associations between High-Sensitivity C-Reactive Protein and Membrane Fluidity of Red Blood Cells in Hypertensive Elderly Men: An Electron Spin Resonance Study. Int. J. Hypertens..

[37] Doudin A., Becker A., Rothenberger A., Meyer T. (2018). Relationship between serum 25-hydroxyvitamin D and red blood cell indices in German adolescents. Eur. J. Pediatr..

[38] Saha A.K., Schmidt B.R., Wilhelmy J., Nguyen V., Abugherir A., Do J.K., Nemat-Gorgani M., Davis R.W., Ramasubramanian A.K. (2019). Red blood cell deformability is diminished in patients with Chronic Fatigue Syndrome. Clin. Hemorheol. Microcirc..

[39] MacLaughlin J., Holick M.F. (1985). Aging decreases the capacity of human skin to produce vitamin D3. J. Clin. Investig..

[40] Van der Wielen R.P., De Groot L.C.P.G.M., Van Staveren W.A., Löwik M.R.H., Van den Berg H., Haller J., Moreiras O. (1995). Serum vitamin D concentrations among elderly people in Europe. Lancet.

[41] Chapuy M.C., Schott A.M., Garnero P., Hans D., Delmas P.D., Meunier P.J. (1996). Healthy elderly French women living at home have secondary hyperparathyroidism and high bone turnover in winter. EPIDOS Study Group. J. Clin. Endocrinol. Metab..

[42] Aguado P., del Campo M.T., Garcés M.V., González-Casaús M.L., Bernad M., Gijón-Baños J., Martín Mola E., Torrijos A., Martínez M.E. (2000). Low vitamin D levels in outpatient postmenopausal women from a rheumatology clinic in Madrid, Spain: Their relationship with bone mineral density. Osteoporos. Int..

[43] Romagnoli E., Caravella P., Scarnecchia L., Martinez P., Minisola S. (1999). Hypovitaminosis D in an Italian population of healthy subjects and hospitalized patients. Br. J. Nutr..

[44] Reginster J.Y., Halkin V., Henrotin Y., Gosset C. (1999). Treatment of osteoporosis: Role of bone-forming agents. Osteoporos. Int..

[45] Eriksen E.F., Glerup H. (2002). Vitamin D deficiency and aging: Implications for general health and osteoporosis. Biogerontology.

[46] Chan R., Woo J. (2011). The value of vitamin D supplementation in older people. Nutr. Ther. Metab..

[47] Holick M.F. (2011). Vitamin D: A d-lightful solution for health. J. Investig. Med..

[48] Cesari M., Incalzi R.A., Zamboni V., Pahor M. (2011). Vitamin D hormone: A multitude of actions potentially influencing the physical function decline in older persons. Geriatr. Gerontol. Int..

[49] Havdahl A., Mitchell R., Paternoster L., Davey Smith G. (2019). Investigating causality in the association between vitamin D status and self-reported tiredness. Sci. Rep..

[50] Engberg I., Segerstedt J., Waller G., Wennberg P., Eliasson M. (2017). Fatigue in the general population-associations to age, sex, socioeconomic status, physical activity, sitting time and self-rated health: The northern Sweden MONICA study 2014. BMC Public Health.

[51] Ginde A.A., Liu M.C., Camargo C.A. (2009). Demographic Differences and Trends of Vitamin D Insufficiency in the US Population, 1988–2004. Arch. Intern. Med..

[52] Goedendorp M.M., Knoop H., Schippers G.M., Bleijenberg G. (2009). The lifestyle of patients with chronic fatigue syndrome and the effect on fatigue and functional impairments. J. Hum. Nutr. Diet..

[53] Taylor B.A., Lorson L., White C.M., Thompson P.D. (2017). Low vitamin D does not predict statin associated muscle symptoms but is associated with transient increases in muscle damage and pain. Atherosclerosis.

[54] Bhat M., Ismail A. (2015). Vitamin D treatment protects against and reverses oxidative stress induced muscle proteolysis. J. Steroid Biochem. Mol. Biol..

[55] Oliveri B., Plantalech L., Bagur A., Wittich A.C., Rovai G., Pusiol E., López Giovanelli J., Ponce G., Nieva A., Chaperón A. (2004). High prevalence of vitamin D insufficiency in healthy elderly people living at home in Argentina. Eur. J. Clin. Nutr..

[56] Bettica P., Bevilacqua M., Vago T., Norbiato G. (1999). High prevalence of hypovitaminosis D among free-living postmenopausal women referred to an osteoporosis outpatient clinic in northern Italy for initial screening. Osteoporos. Int..

